# Redetermination of bis­(*O*,*O*′-diethyl dithio­phosphato-κ^2^ *S*,*S*′)nickel(II)

**DOI:** 10.1107/S160053680901767X

**Published:** 2009-05-20

**Authors:** Damian Paliwoda, Jarosław Chojnacki, Anna Mietlarek-Kropidłowska, Barbara Becker

**Affiliations:** a Department of Inorganic Chemistry, Chemical Faculty, Gdańsk University of Technology, 11/12 G. Narutowicza Str., 80-233 Gdańsk, Poland

## Abstract

The centrosymmetric title complex, [Ni{S_2_P(OC_2_H_5_)_2_}_2_], has been redetermined using area-detector data. The central Ni(S_2_P)_2_ core is essentially planar and confirms the early results of McConnell & Kastalsky [*Acta Cryst.* (1967), **22**, 853–859] based on multiple film technique data. In the title structure, the standard uncertainty values are approximately seven times lower and all H-atom positions are calculated. A pair of short symmetry-related H⋯H contacts with distances of 2.33 Å is observed in the crystal structure.

## Related literature

For the syntheses and structure of a series of homologous Ni(S_2_P{O*R*}_2_)_2 _complexes, see: *R* = Me: Kastalsky & McConnell (1969 ▶); *R* = Et: Fernando & Green (1967 ▶); McConnell & Kastalsky (1967 ▶); *R* = Pr and *R* = ^*i*^Bu: Ivanov *et al.* (2004 ▶); *R* = ^*i*^Pr: Tkachev & Atovmyan (1976 ▶); Hoskins & Tiekink (1985 ▶). For complexes with sulfur-rich kernel-bearing silanethiol­ato and dithio­carbamato ligands, see: Kropidłowska *et al.* (2008 ▶). For hydrogen bonds, see: Steiner & Desiraju (1998 ▶).
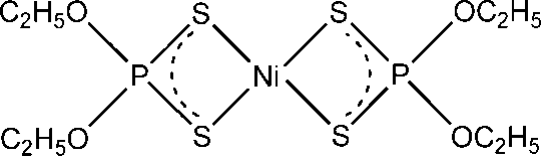


## Experimental

### 

#### Crystal data

[Ni(C_4_H_10_O_2_PS_2_)_2_]
*M* *_r_* = 429.13
Monoclinic, 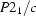

*a* = 10.4810 (4) Å
*b* = 10.2777 (3) Å
*c* = 8.7541 (3) Å
β = 102.820 (3)°
*V* = 919.49 (6) Å^3^
*Z* = 2
Mo *K*α radiation
μ = 1.69 mm^−1^
*T* = 295 K
0.41 × 0.34 × 0.09 mm

#### Data collection

Oxford Diffraction KM-4-CCD diffractometer
Absorption correction: multi-scan (*CrysAlis RED*; Oxford Diffraction, 2008 ▶) *T*_min_ = 0.530, *T*_max_ = 0.853
7073 measured reflections
2012 independent reflections
1768 reflections with *I* > 2σ(*I*)
*R*_int_ = 0.016

#### Refinement

*R*[*F* ^2^ > 2σ(*F* ^2^)] = 0.031
*wR*(*F* ^2^) = 0.083
*S* = 1.09
2012 reflections
91 parameters
H-atom parameters constrained
Δρ_max_ = 0.30 e Å^−3^
Δρ_min_ = −0.32 e Å^−3^

### 

Data collection: *CrysAlis CCD* (Oxford Diffraction, 2008 ▶); cell refinement: *CrysAlis RED* (Oxford Diffraction, 2008 ▶); data reduction: *CrysAlis RED*; program(s) used to solve structure: *SHELXS97* (Sheldrick, 2008 ▶); program(s) used to refine structure: *SHELXL97* (Sheldrick, 2008 ▶); molecular graphics: *ORTEP-3 for Windows* (Farrugia, 1997 ▶) and *Mercury* (Macrae *et al.*, 2006 ▶); software used to prepare material for publication: *WinGX* (Farrugia, 1999 ▶).

## Supplementary Material

Crystal structure: contains datablocks global, I. DOI: 10.1107/S160053680901767X/si2169sup1.cif

Structure factors: contains datablocks I. DOI: 10.1107/S160053680901767X/si2169Isup2.hkl

Additional supplementary materials:  crystallographic information; 3D view; checkCIF report

## Figures and Tables

**Table:** 

Ni1—S2	2.2253 (6)
Ni1—S1	2.2254 (6)
P1—S1	1.9876 (8)
P1—S2	1.9890 (8)

**Table:** 

S2—Ni1—S1	88.41 (2)
S1—P1—S2	102.58 (3)
P1—S1—Ni1	84.50 (3)
P1—S2—Ni1	84.47 (3)

**Fig. 1.**
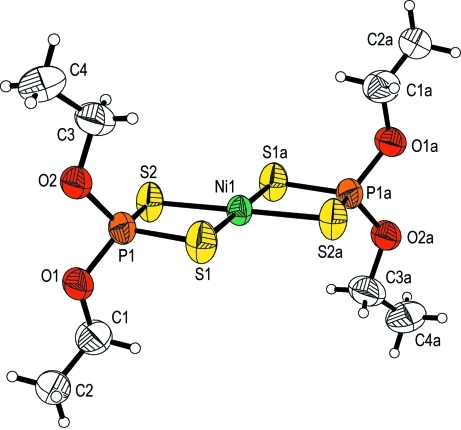
Molecular structure and atom-numbering scheme for [Ni(S2P{OC2H5}2)2] with displacement ellipsoids drawn at 50% probability level. H atoms are represented as arbitrary circles.

**Fig. 2.**
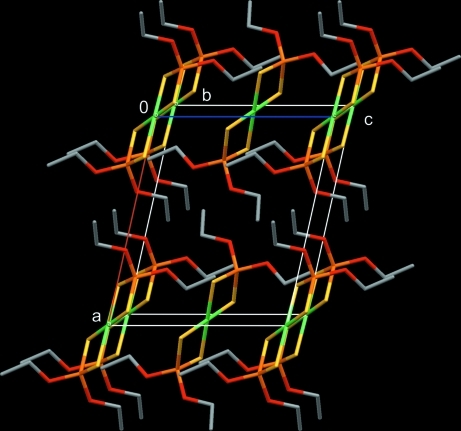
Crystal packing for [Ni(S2P{OC2H5}2)2].

**Fig. 3.**
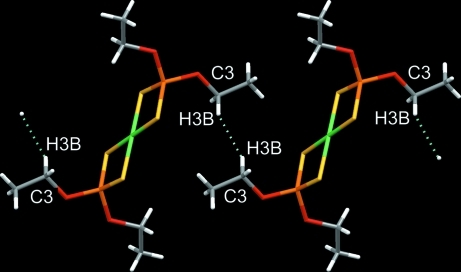
Short intermolecular C–H···H–C contacts (view along b axis).

## supplementary crystallographic information

### Comment

We are interested in metal complexes with sulfur rich kernel (Kropidłowska, Chojnacki, *et al.*, 2008). Recently, we turned our attention to dithiophosphates and among others to the nickel(II) complexes with widely used diethyldithiophosphate ligand (DEDTP). We prepared the title Ni(DEDTP)_2_ complex and redetermined its structure. The nickel atom of Ni(DEDTP)_2_ molecule (Fig. 1) occupies a crystallographic inversion centre and shows a square planar geometry, which was also observed previously by Fernando & Green (Fernando & Green, 1967; NIETHP01) and McConnell & Kastalsky (McConnell & Kastalsky, 1967; NIETHP), who used multiple film technique data for the structure determination. For NIETHP01 (*R* = 15.7%) the average distances and angles within four-membered NiS_2_P chelate ring were given as Ni—S 2.21 (1) Å, P—S 1.97 (2) Å, S1—Ni—S2 88°, and S1—P—S2 103°. More precise values were reported for NIETHP (*R* = 11.5%) with the following distances and angles within chelate NiS_2_P ring: Ni—S1 2.230 (4) Å, Ni—S2 2.236 (4) Å, S1—P 1.986 (6) Å, S2—P 1.993 (5), S1—Ni—S2 88.5 (1)° and S1—P—S2 103.1 (2)°.

These may be compared to the present data - respective values are given in Table 2. Note almost identical bond lengths for the pairs of Ni–*S* and *S*–P bonds. The redetermination was done at room temperature and the structure was refined with a crystallographic reliability of *R*=3.06%. Although the overal picture did not change significantly, the precision of the present data is much higher. Lighter atoms (C, O) are well fixed, standard uncertainty values are approximately seven times lower than those of reported NIETHP and NIETHP01 structures and all H-atom positions are calculated.

In general, Ni–S bond lengths in several nickel(II) dialkyldithiophosphates, [Ni(S_2_P{OR}_2_)_2_] are quite similar. Those with *R* = Me (2.219 (2) and 2.225 (2) Å) (Kastalsky & McConnell, 1969, DMTPON), Pr (2.2255 (6) - 2.2344 (5) Å) (Ivanov *et al.*, 2004, IBAQAE), *^i^*Pr (2.216 (1) and 2.227 (1) Å) (Tkachev & Atovmyan, 1976, IPDTPN, Hoskins & Tiekink, 1985, IPDTPN01) and *^i^*Bu (2.218 (1) - 2.231 (1) Å) (Ivanov *et al.*, 2004, IBAQEI) are within 2.218 - 2.235 Å range. Also S–P bond lengths change only slightly and all are within 1.98 - 2.00 Å. Crystals of Ni(DEDTP)_2_ consist of discrete units of the complex (Fig. 2) and besides short intermolecular C3—H3B ··· H3B—C3 contact between the symmetry related molecules (H···H distance equals 2.33 Å, see Fig. 3) there are no other interactions. Although the above mentioned contact is comparable with the sum of two hydrogen atoms van der Waals radii (2.4 Å) there is no reason to consider it as a weak hydrogen bond (Steiner & Desiraju, 1998). None of the aforementioned nickel(II) dialkyldithiophosphates show such C–H···H–C nonbonding interactions although for di(iso-butyl)dithiophosphate (Ivanov *et al.*, 2004) the existence of weak C–H···S hydrogen bonds may be envisaged.

### Experimental

Nickel chloride, NiCl_2_×6H_2_O (0.60 g; 0.0025 mol; POCh) was dissolved in 30 ml me thanol/water (10/1, *v*/*v*) and added dropwise to the solution of ammonium salt of diethyldithiophosphate (1.02 g; 0.005 mol; Aldrich) in 20 ml me thanol/water (10/1, *v*/*v*). The mixture was stirred vigorously for one hour. The solution was then filtered off and the filtrate was left for crystallization at room temperature. After one day well shaped, violet prismatic crystals suitable for X-ray analysis were collected. Then, the mother liquor was concentrated and after few days more product was isolated. The overall yield was *c.a.* ~90%.

### Refinement

All H atoms were placed in calculated positions and refined as riding on their carrier atoms with respective *U*_iso_(H) values: C—H = 0.96 Å (CH_3_) and *U*_iso_(H) = 1.5 *U*_eq_(C), C—H = 0.97 Å (CH_2_) and *U*_ĩso_(H) = 1.2 *U*_eq_(C).

### Crystal data

**Table:**

[Ni(C_4_H_10_O_2_PS_2_)_2_]	*F*(000) = 444
*M**_r_* = 429.13	*D*_x_ = 1.55 Mg m^−^^3^
Monoclinic, *P*2_1_/*c*	Melting point: 378 K
Hall symbol: -P 2ybc	Mo *K*α radiation, λ = 0.71073 Å
*a* = 10.4810 (4) Å	Cell parameters from 5670 reflections
*b* = 10.2777 (3) Å	θ = 2.0–32.3°
*c* = 8.7541 (3) Å	µ = 1.69 mm^−^^1^
β = 102.820 (3)°	*T* = 295 K
*V* = 919.49 (6) Å^3^	Prism, violet
*Z* = 2	0.41 × 0.34 × 0.09 mm

### Data collection

**Table:**

Oxford Diffraction KM-4-CCD diffractometer	2012 independent reflections
graphite	1768 reflections with *I* > 2σ(*I*)
Detector resolution: 8.1883 pixels mm^-1^	*R*_int_ = 0.016
ω (0.75° width) scans	θ_max_ = 27.0°, θ_min_ = 2.8°
Absorption correction: multi-scan (*CrysAlis RED*; Oxford Diffraction, 2008)	*h* = −13→13
*T*_min_ = 0.530, *T*_max_ = 0.853	*k* = −7→13
7073 measured reflections	*l* = −11→11

### Refinement

**Table:**

Refinement on *F*^2^	Secondary atom site location: difference Fourier map
Least-squares matrix: full	Hydrogen site location: inferred from neighbouring sites
*R*[*F*^2^ > 2σ(*F*^2^)] = 0.031	H-atom parameters constrained
*wR*(*F*^2^) = 0.083	*w* = 1/[σ^2^(*F*_o_^2^) + (0.0441*P*)^2^ + 0.3217*P*] where *P* = (*F*_o_^2^ + 2*F*_c_^2^)/3
*S* = 1.09	(Δ/σ)_max_ = 0.001
2012 reflections	Δρ_max_ = 0.30 e Å^−^^3^
91 parameters	Δρ_min_ = −0.32 e Å^−^^3^
0 restraints	Extinction correction: *SHELXL97* (Sheldrick, 2008), Fc^*^=kFc[1+0.001xFc^2^λ^3^/sin(2θ)]^-1/4^
Primary atom site location: structure-invariant direct methods	Extinction coefficient: 0.027 (2)

### Special details

**Table:**

Experimental. Oxford Diffraction Ltd., 2008. Empirical absorption correction using spherical harmonics, implemented in SCALE3 ABSPACK scaling algorithm.
Geometry. All e.s.d.'s (except the e.s.d. in the dihedral angle between two l.s. planes) are estimated using the full covariance matrix. The cell e.s.d.'s are taken into account individually in the estimation of e.s.d.'s in distances, angles and torsion angles; correlations between e.s.d.'s in cell parameters are only used when they are defined by crystal symmetry. An approximate (isotropic) treatment of cell e.s.d.'s is used for estimating e.s.d.'s involving l.s. planes.
Refinement. Refinement of *F*^2^ against ALL reflections. The weighted *R*-factor *wR* and goodness of fit *S* are based on *F*^2^, conventional *R*-factors *R* are based on *F*, with *F* set to zero for negative *F*^2^. The threshold expression of *F*^2^ > σ(*F*^2^) is used only for calculating *R*-factors(gt) *etc*. and is not relevant to the choice of reflections for refinement. *R*-factors based on *F*^2^ are statistically about twice as large as those based on *F*, and *R*- factors based on ALL data will be even larger.

### Fractional atomic coordinates and isotropic or equivalent isotropic displacement parameters (Å^2^)

**Table:**

	*x*	*y*	*z*	*U*_iso_*/*U*_eq_	
Ni1	0	1	0	0.04253 (14)	
P1	−0.23354 (5)	0.94137 (6)	0.09934 (7)	0.04844 (17)	
S1	−0.07705 (6)	0.82989 (6)	0.10854 (9)	0.0633 (2)	
S2	−0.18740 (6)	1.10251 (6)	−0.00212 (8)	0.06062 (19)	
O1	−0.36674 (16)	0.87744 (19)	0.01719 (18)	0.0621 (4)	
O2	−0.26962 (16)	0.96353 (18)	0.26213 (18)	0.0583 (4)	
C1	−0.3979 (3)	0.8474 (3)	−0.1498 (3)	0.0745 (8)	
H1A	−0.3223	0.8095	−0.1799	0.089*	
H1B	−0.4217	0.9263	−0.2102	0.089*	
C2	−0.5078 (3)	0.7549 (3)	−0.1810 (4)	0.0786 (8)	
H2A	−0.4839	0.6779	−0.1192	0.118*	
H2B	−0.5282	0.7322	−0.2901	0.118*	
H2C	−0.5829	0.7942	−0.1539	0.118*	
C3	−0.1754 (3)	1.0198 (4)	0.3917 (4)	0.0835 (9)	
H3A	−0.1529	1.1071	0.3648	0.1*	
H3B	−0.0962	0.9678	0.4135	0.1*	
C4	−0.2321 (4)	1.0239 (3)	0.5302 (3)	0.0873 (9)	
H4A	−0.3118	1.0732	0.507	0.131*	
H4B	−0.1713	1.0642	0.6153	0.131*	
H4C	−0.2503	0.9369	0.5592	0.131*	

### Atomic displacement parameters (Å^2^)

**Table:**

	*U*^11^	*U*^22^	*U*^33^	*U*^12^	*U*^13^	*U*^23^
Ni1	0.0394 (2)	0.0400 (2)	0.0513 (2)	−0.00037 (13)	0.01666 (15)	0.00300 (14)
P1	0.0433 (3)	0.0526 (3)	0.0531 (3)	−0.0052 (2)	0.0188 (2)	0.0003 (2)
S1	0.0587 (4)	0.0458 (3)	0.0936 (5)	0.0031 (2)	0.0342 (3)	0.0150 (3)
S2	0.0514 (3)	0.0525 (3)	0.0849 (4)	0.0098 (2)	0.0300 (3)	0.0166 (3)
O1	0.0524 (9)	0.0873 (12)	0.0500 (8)	−0.0183 (8)	0.0184 (7)	−0.0112 (8)
O2	0.0519 (9)	0.0756 (10)	0.0502 (8)	−0.0125 (8)	0.0171 (7)	−0.0067 (8)
C1	0.0777 (18)	0.097 (2)	0.0518 (13)	−0.0140 (16)	0.0204 (12)	−0.0100 (13)
C2	0.0576 (15)	0.100 (2)	0.0758 (17)	−0.0038 (15)	0.0089 (12)	−0.0278 (16)
C3	0.0717 (18)	0.113 (2)	0.0635 (16)	−0.0253 (17)	0.0093 (13)	−0.0236 (16)
C4	0.108 (3)	0.093 (2)	0.0587 (16)	−0.0009 (19)	0.0146 (16)	−0.0152 (15)

### Geometric parameters (Å, °)

**Table:**

Ni1—S2	2.2253 (6)	C1—C2	1.472 (4)
Ni1—S2^i^	2.2253 (6)	C1—H1A	0.97
Ni1—S1	2.2254 (6)	C1—H1B	0.97
Ni1—S1^i^	2.2254 (6)	C2—H2A	0.96
Ni1—P1	2.8382 (5)	C2—H2B	0.96
Ni1—P1^i^	2.8382 (5)	C2—H2C	0.96
P1—O1	1.5660 (17)	C3—C4	1.464 (4)
P1—O2	1.5700 (16)	C3—H3A	0.97
P1—S1	1.9876 (8)	C3—H3B	0.97
P1—S2	1.9890 (8)	C4—H4A	0.96
O1—C1	1.459 (3)	C4—H4B	0.96
O2—C3	1.449 (3)	C4—H4C	0.96
			
S2—Ni1—S2^i^	180	C1—O1—P1	121.83 (15)
S2—Ni1—S1	88.41 (2)	C3—O2—P1	120.60 (17)
S2^i^—Ni1—S1	91.59 (2)	O1—C1—C2	108.3 (2)
S2—Ni1—S1^i^	91.59 (2)	O1—C1—H1A	110
S2^i^—Ni1—S1^i^	88.41 (2)	C2—C1—H1A	110
S1—Ni1—S1^i^	180	O1—C1—H1B	110
S2—Ni1—P1	44.230 (19)	C2—C1—H1B	110
S2^i^—Ni1—P1	135.770 (19)	H1A—C1—H1B	108.4
S1—Ni1—P1	44.194 (19)	C1—C2—H2A	109.5
S1^i^—Ni1—P1	135.81 (2)	C1—C2—H2B	109.5
S2—Ni1—P1^i^	135.770 (19)	H2A—C2—H2B	109.5
S2^i^—Ni1—P1^i^	44.230 (19)	C1—C2—H2C	109.5
S1—Ni1—P1^i^	135.81 (2)	H2A—C2—H2C	109.5
S1^i^—Ni1—P1^i^	44.194 (19)	H2B—C2—H2C	109.5
P1—Ni1—P1^i^	180	O2—C3—C4	109.2 (3)
O1—P1—O2	96.20 (9)	O2—C3—H3A	109.8
O1—P1—S1	114.85 (8)	C4—C3—H3A	109.8
O2—P1—S1	114.18 (8)	O2—C3—H3B	109.8
O1—P1—S2	115.13 (8)	C4—C3—H3B	109.8
O2—P1—S2	114.58 (8)	H3A—C3—H3B	108.3
S1—P1—S2	102.58 (3)	C3—C4—H4A	109.5
O1—P1—Ni1	133.63 (6)	C3—C4—H4B	109.5
O2—P1—Ni1	130.17 (6)	H4A—C4—H4B	109.5
S1—P1—Ni1	51.30 (2)	C3—C4—H4C	109.5
S2—P1—Ni1	51.30 (2)	H4A—C4—H4C	109.5
P1—S1—Ni1	84.50 (3)	H4B—C4—H4C	109.5
P1—S2—Ni1	84.47 (3)		

Symmetry codes: (i) −*x*, −*y*+2, −*z*.
